# Direct Epicardial Mapping and Ablation Revealing Three‐Dimensional Roof‐Dependent Macroreentrant Atrial Tachycardia Breaking Into Bachmann's Bundle

**DOI:** 10.1002/ccr3.70391

**Published:** 2025-04-04

**Authors:** Yuya Suzuki, Kaoru Takami, Tomomi Akita, Tatsuru Nomura, Koji Fukuzawa, Akihiro Yoshida

**Affiliations:** ^1^ Department of Cardiology Kita‐Harima Medical Center Ono Japan; ^2^ Department of Clinical Engineering Kita‐Harima Medical Center Ono Japan; ^3^ Section of Arrhythmia, Division of Cardiovascular Medicine, Department of Internal Medicine Kobe University Graduate School of Medicine Kobe Japan

**Keywords:** ablation, atrial tachycardia, Bachmann's bundle, Epicardial mapping, roof‐dependent

## Abstract

Catheter ablation for macroreentrant atrial tachycardia (AT) that recurs after atrial fibrillation (AF) ablation is occasionally difficult because of epicardial connections. Here, we report the case of a patient who underwent ablation for recurrent AT after AF ablation. Endocardial mapping of the left atrium (LA) showed a centrifugal pattern from the base of the appendage, and the LA roof showed discontinuous activation from the posterior LA. Direct epicardial mapping was performed, revealing the roof‐dependent macroreentrant AT using the epicardial connection from the LA posterior to the Bachmann bundle. The entrainment pacing from the exit site of the epicardium was within the tachycardia circuit, and epicardial ablation successfully terminated the AT. Epicardial mapping and ablation can improve the procedural success rate of reentrant AT recurrence after endocardial ablation.

**Abbreviations**
AF: atrial fibrillation
AT: atrial tachycardia
LA: left atrium

**Summary**

- Direct epicardial mapping and ablation are valuable options for managing refractory, recurrent atrial tachycardia following endocardial roof line ablation, particularly when epicardial connections such as Bachmann's bundle are implicated.

## 1 Introduction

Catheter ablation of atrial fibrillation (AF) often results in the recurrence of macroreentrant atrial tachycardia (AT) [1]. Macroreentrant AT is induced by anatomic obstacles and arrhythmogenic substrate created by prior ablation, including isolated pulmonary veins [2, 3]. Although high‐density mapping is useful for identifying the complete AT circuit, the acute success of AT ablation is limited, and recurrences are not uncommon [4]. Recently, epicardial connections (including Bachmann's bundle, the coronary sinus, the Marshall ligament, and the septopulmonary bundle) have been reported to play crucial roles in the development of AT. Because of the thickness of the atrium in the roof area, non‐transmural lesions caused by previous endocardial ablation may lead to epicardial‐related AT. Endocardial mapping alone cannot reveal the entire circuit of the AT using an epicardial connection. Direct epicardial mapping and ablation targeting recurrent AT involving the epicardial connection after failed endocardial ablation may improve procedural success [5]. Here, we report a case of successful epicardial ablation of roof‐dependent macroreentrant AT conducted through the epicardial Bachmann's bundle following endocardial ablation of the roof area.

### 1.1 Case History and Examination

**FIGURE 1:**
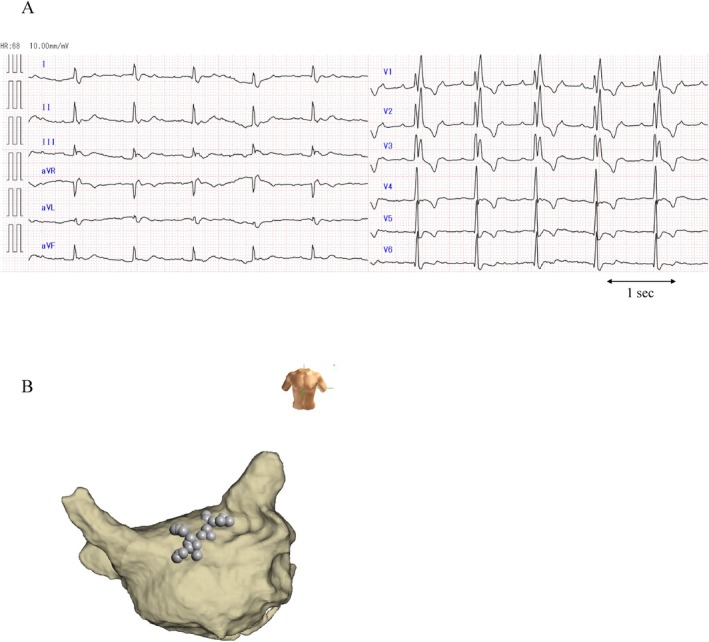
12‐Lead electrocardiogram (ECG) of atrial tachycardia observed at admission and 3D image of the left atrium highlighting the roof area ablation points from the previous procedure. (A) A 12‐lead ECG of atrial tachycardia (AT) in a patient with recurrent AT after the fourth catheter ablation session. The ECG showed isoelectric P waves in the inferior leads and positive P waves in lead V1. The AT cycle length was 300 ms, with 3:1 atrioventricular conduction. (B) Three‐dimensional (3D) mapping of the left atrial (LA) endocardium using the NavX system showing the ablation points from the previous roofline ablation session. The tag marked the ablation sites on the roof area from the previous session onward.

An 82‐year‐old male with palpitations was admitted to our hospital. A baseline 12‐lead electrocardiogram revealed AT (Figure 1A). He had previously undergone extensive endocardial radiofrequency catheter ablation four times for paroxysmal AF and AT recurrence, which included ablation of the pulmonary vein, superior vena cava, mitral isthmus, and cavotricuspid isthmus. Additionally, roof line ablation was performed to modify the AF rotor in the roof (Figure 1B). However, perimitral atrial flutter was induced, and mitral isthmus ablation was unsuccessful. Although electrical cardioversion was performed, AT recurred 3 h after the procedure. Moreover, AT was refractory to anti‐arrhythmic drugs. Three months later, a fifth ablation was performed using an epicardial approach. Because the AT was refractory to endocardial catheter ablation, we performed both endocardial and epicardial ablations.

### 1.2 Differential Diagnosis, Investigations and Treatment

**FIGURE 2:**
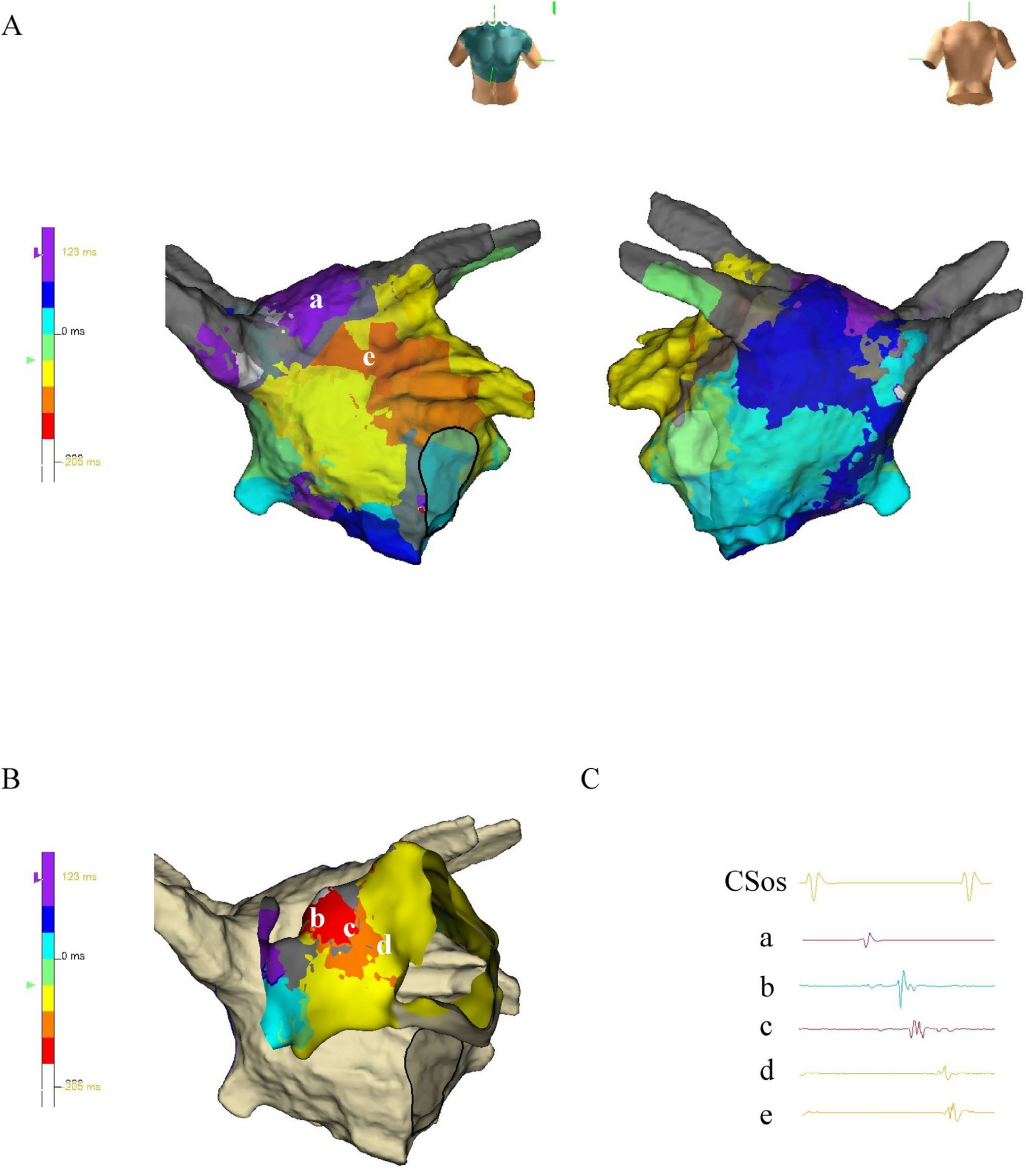
Endocardial and epicardial activation mapping of atrial tachycardia. (A) Activation mapping of the left atrial (LA) endocardium during atrial tachycardia (AT). (B) Activation mapping of the left atrial epicardium and endocardium during AT tachycardia (C), Endocardial and epicardial recordings of the AT. (a) and (e) LA endocardium; (b), (c), and (d) LA epicardium.

Electroanatomical mapping was performed using an EnSite system (Abbott Laboratories, IL, USA) and a high‐resolution mapping catheter (Advisor HD‐Grid, Abbott Laboratories). Initial endocardial mapping of the LA revealed a centrifugal activation pattern, with the earliest atrial activation site located in the roof area near the LA appendage, where Bachmann's bundle is connected to the LA endocardium from the epicardium. The LA activation time was 227 ms compared to the AT cycle length of 328 ms. The missing segments of 101 ms on endocardial mapping were thought to be involved in epicardial conduction (Figure 2A). Additional mapping of the aortic cusp and pulmonary artery could not record epicardial electrograms. To clarify this, direct epicardial mapping was performed using a subxiphoid anterior approach in addition to endocardial mapping. Combined endocardial and epicardial mapping revealed a roof‐dependent macroreentrant AT involving an epicardial connection from the LA posterior wall to the Bachmann's bundle. The opposite endocardial roof showed a scar and a discontinuous activation pattern (Figure 2B,C). The post‐pacing interval from the epicardial exit site (green tag) was 17 ms, indicating less than 20 ms, suggesting the epicardial site was within the tachycardia circuit. The AT wavefront propagated from the endocardial LA highly posterior to the epicardial exit site, broke into Bachmann's bundle, and then reconnected to the endocardium near the LA appendage.

### 1.3 Outcome and Follow‐Up

**FIGURE 3:**
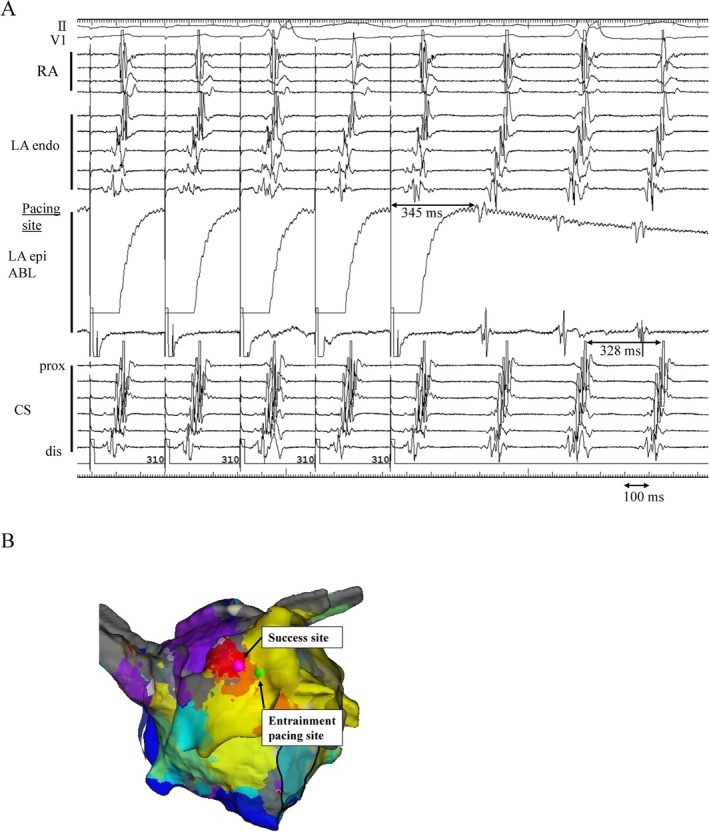
Good entrainment response and successful ablation point. (A) Entrainment pacing from the epicardial site (green tag) using an ablation catheter demonstrated a good entrainment response, with a post‐pacing interval – tachycardia cycle length = 17 ms ≤ 20 ms. (B) Successful ablation site (pink tag) and entrainment pacing site (green tag) in the epicardium. ABL, Ablation catheter; CS, coronary sinus; dis, distal; endo, endocardium; epi, epicardium; LA, left atrium; prox, proximal; RA, right atrium.

Ablation at the epicardial breakthrough site (pink tag) immediately terminated AT (Figure 3). Following AT termination, programmed atrial stimulation induced perimitral flutter via the coronary sinus, where the previously ablated endocardial mitral isthmus line remained disconnected. Epicardial mapping of the mitral isthmus line revealed no signal activation. Ablation within the coronary sinus terminated the perimitral flutter, and no further AT was induced. Postoperatively, no complications were associated with the endocardial or epicardial ablation. Six months after the procedure, the patient has remained free of AT recurrence, but further follow‐up is required.

## 2 Discussion

For the first time, we successfully demonstrated a three‐dimensional roof‐dependent macroreentrant AT involving the epicardial Bachmann's bundle using direct epicardial mapping.

Bachmann's bundle is a wide muscular band that extends epicardially, bifurcates, and passes through the right atrial and LA appendages. After traversing the interatrial groove, it continues leftward over the anterosuperior part of the LA (the roof area) [6]. Bachmann's bundle is commonly involved in the epicardial circuits of biatrial tachycardia and can serve as a target for ablation [7]. However, reports on its role in other types of macroreentrant AT remain limited. Yamaoka et al. described a left atrial macroreentrant AT mediated by Bachmann's bundle, which rotated clockwise along the LA anterior wall using the epicardial bundle [8]. Similarly, Nakamura et al. reported a roof‐dependent AT involving an epicardial circuit that mimicked a posterior focal AT [9]. Furthermore, Yorgun et al. reported a perimitral flutter involving the Bachmann's bundle [10]. There were also previous reports on the long‐term safety and effectiveness of epicardial ablation for recurrent AT or AF after prior endocardial ablation [11, 12].

The roof area of the LA is relatively thicker because of the epicardial myofibers of Bachmann's bundle and septopulmonary bundles [13, 14]. Therefore, non‐transmural lesions from previous endocardial ablation might lead to the creation of a substrate for the epicardial‐related AT, just as a three‐dimensional reentrant ventricular tachycardia, via endocardial, epicardial, and intramural substrates, has been reported [15]. At the second AT procedure, epicardial connections were involved in almost half of recurrent ATs [16], and identified in approximately 30% of roof‐dependent macroreentrant ATs. Baskovski et al. reported that the endocardial ablation of the breakthrough site of the epicardial ATs. The ablation for the Roof‐dependent AT involving the epicardial Backmann's bundle is difficult to diagnose and was often effective in terminating the arrhythmia during the session, but a quarter of those patients recurred during follow‐up [17]. Another approach was ablation of the isthmus of roof‐dependent AT located between the left and right pulmonary veins. If the post pacing interval by entrainment pacing from LA posterior matched the AT cycle length, AT might have stopped by bottom line between both inferior pulmonary veins. Nowadays, the additional mapping of the right and left pulmonary artery branches (called “natural surface” epicardial mapping) is focused on because of the highly invasive procedure of direct epicardial mapping. “Natural surface” epicardial mapping can sometimes enable the recording of the electrogram of the Bachmann's bundle and entrain the epicardial Bachman's bundle [18]. In this case, the potential of Bachmann's bundle was unfortunately not possible to record from the pulmonary arteries.

In our case, we provided direct evidence of a roof‐dependent macroreentrant AT using Backmann's bundle through direct epicardial mapping. This approach, combined with endocardial mapping, is critical for revealing the three‐dimensional structure of the circuit and enabling effective treatment. Roof line ablation was previously performed to modify the AF rotor in the roof area but was insufficient to resolve the AT. Our findings suggest that direct epicardial mapping and ablation are valuable options for managing refractory, recurrent AT following endocardial roof line ablation, particularly when epicardial connections such as the Bachmann's bundle are implicated.

## 3 Limitations

First, the sample size was limited. Therefore, a larger sample size is required. Second, the follow‐up periods were 6 months after the procedure, and further follow‐up is needed.

## 4 Conclusion

Epicardial and endocardial mapping identified three‐dimensional roof‐dependent macroreentrant AT recurrence following endocardial roof line ablation. Epicardial ablation was performed at the breakthrough site, where conduction emerged from the endocardial LA posterior, successfully terminating the AT. Incorporating epicardial mapping and ablation can enhance procedural success in patients with recurrent AT following endocardial ablation.

## Author Contributions

**Yuya Suzuki:** investigation, writing – original draft. **Kaoru Takami:** methodology, writing – review and editing. **Tomomi Akita:** data curation, visualization. **Tatsuru Nomura:** formal analysis, visualization. **Koji Fukuzawa:** investigation, validation. **Akihiro Yoshida:** investigation, writing – review and editing.

## Disclosure

The arrhythmia department of Kobe University Graduate School of Medicine was supported by endowments from Abbott Japan, Boston Scientific Japan, and Medtronic Japan. Dr. Fukuzawa is part of this section. Dr. Fukuzawa received a scholarship from Biotronik (Japan). The authors report no relationships relevant to the content of this study.

## Ethics Statement

Ethical approval was not required for this case report as it describes the clinical management of the patient and does not involve any experimental interventions. All clinical procedures were performed in accordance with institutional guidelines and the Declaration of Helsinki.

## Consent

Written informed consent was obtained from the patient for the publication of this case report and any accompanying images.

## Conflicts of Interest

The authors declare no conflicts of interest.

## Data Availability

Data sharing is not applicable to this article as no datasets were generated or analyzed during the current study.
